# Height-diameter allometry and above ground biomass in tropical montane forests: Insights from the Albertine Rift in Africa

**DOI:** 10.1371/journal.pone.0179653

**Published:** 2017-06-15

**Authors:** Gérard Imani, Faustin Boyemba, Simon Lewis, Nsharwasi Léon Nabahungu, Kim Calders, Louis Zapfack, Bernard Riera, Clarisse Balegamire, Aida Cuni-Sanchez

**Affiliations:** 1 Biology Department, Université Officielle de Bukavu, Bukavu, DR Congo; 2 Plant Department, Université de Kisangani, Kisangani, DR Congo; 3 Department of Geography, University College London, London, United Kingdom; 4 School of Geography, University of Leeds, Leeds, United Kingdom; 5 Soil Laboratory, International Institute of Tropical Agriculture/Kalambo site, Kalambo, DR Congo; 6 Earth Observation, Climate and Optical Group, National Physical Laboratory, Teddington, Middlesex, United Kingdom; 7 Plant Biology and physiology Department, University of Yaoundé 1, Yaoundé, Cameroun; 8 Laboratoire d’Ecologie générale, Museum National d’Histoire Naturelle, Brunoy, France; 9 Centre de Recherche en Sciences Naturelles de Lwiro, Lwiro, DR Congo; Tennessee State University, UNITED STATES

## Abstract

Tropical montane forests provide an important natural laboratory to test ecological theory. While it is well-known that some aspects of forest structure change with altitude, little is known on the effects of altitude on above ground biomass (AGB), particularly with regard to changing height-diameter allometry. To address this we investigate (1) the effects of altitude on height-diameter allometry, (2) how different height-diameter allometric models affect above ground biomass estimates; and (3) how other forest structural, taxonomic and environmental attributes affect above ground biomass using 30 permanent sample plots (1-ha; all trees ≥ 10 cm diameter measured) established between 1250 and 2600 m asl in Kahuzi Biega National Park in eastern Democratic Republic of Congo. Forest structure and species composition differed with increasing altitude, with four forest types identified. Different height-diameter allometric models performed better with the different forest types, as trees got smaller with increasing altitude. Above ground biomass ranged from 168 to 290 Mg ha^-1^, but there were no significant differences in AGB between forests types, as tree size decreased but stem density increased with increasing altitude. Forest structure had greater effects on above ground biomass than forest diversity. Soil attributes (K and acidity, pH) also significantly affected above ground biomass. Results show how forest structural, taxonomic and environmental attributes affect above ground biomass in African tropical montane forests. They particularly highlight that the use of regional height-diameter models introduces significant biases in above ground biomass estimates, and that different height-diameter models might be preferred for different forest types, and these should be considered in future studies.

## Introduction

Tropical forests play a major role in the global carbon balance [1–4]. With increasing interest in REDD+, an important challenge facing ecologists and foresters is to quantify as precisely as possible the carbon stocks and their fluxes at different spatial scales [5]. Significant milestones have been reached in the last decade thanks to the development of broad-scale remote sensing approaches [2,6,7]. However, local forest biomass estimations based on field measurements commonly represent the foundation for the calibration and validation of remote sensing models [8–11]. As a consequence, uncertainties and errors in local biomass estimations may propagate dramatically to broad-scale forest carbon stock assessment [12–14].

Above ground biomass (AGB) is the major pool of biomass in tropical forests [15]. In the field, the AGB of a tree (or TAGB) is generally estimated either directly or indirectly. In the first case trees are harvested, weighted and dried and weighted again; while in the second case allometric equations are used to estimate biomass from a number of variables, mostly tree diameter, wood density, and sometimes, tree height [16]. It is well known that biomass estimates are more accurate if height is included in these allometric equations [16–19]. However, sampling tree height is difficult in tropical forests [20], especially in montane areas, because of the steep slopes and the difficulty of seeing tree crowns [21]. Therefore, in most cases, tree heights are measured for a number of individuals, and a height-diameter model is applied to estimate the height for the remaining trees [17,22]. Several authors have shown the significant biases in biomass estimates associated with using regional height-diameter models [19,23–26] and highlighted the need for local site specific height-diameter models.

However, there is a debate on which type of model should be selected to build a local site-specific height-diameter model. While some authors support the use of a power law model [18,27] (the one predicted by the metabolic theory of ecology [28,29]), some others support a second order polynomial of the log-log data [30] (the one which considers the saturation of tree height with tree diameter), and others prefer a truly asymptotic model [17,23,31,32] (for further details see [25]). It has been highlighted that the power law model is unrealistic biologically because of the basic assumption of factors limiting tree growth in height but not in diameters [33], and most recent studies have chosen a truly asymptotic model (e.g. Brienen et al. [34]). Among the asymptotic models, Feldpausch et al. [17] found that the Weibull model was the most appropriate for biomass prediction, as it reduces error in small-diameter classes. Banin et al. [35] and Kearsley et al. [19] preferred a nonlinear 3-parameter exponential model. Two recent studies, which included another asymptotic model, the Michaelis-Menten model [25,33], preferred this later one. All these studies focused on tropical lowland forest types, and to our knowledge, the shape of the height-diameter allometry has not been studied in detail in tropical montane forests. Only Ledo et al. [26] assessed one montane forest in Peru and showed that a three-parameter Weibull function was the most accurate height-diameter allometric model.

It is well known that tree height usually decreases with increasing elevation [36]. The rationship between height and diameter is also related to species, climatic, soil characteristics, region and even tree diversity [18,35,37,38]. The presence of bamboo, common in disturbed high montane areas, and wind may also alter forest structure [39–41]. To our knowledge, except Mugasha et al. [21] in Tanzania, no other study has assessed height-diameter relationships in montane forests in Africa. In Tanzania, Mugasha et al. [21] distinguished four vegetation types: lowland forest, montane forest, miombo woodland and Acacia savanna. The height-diameter allometric model differed according to vegetation type [21]. In montane forests, for example, these authors showed that the exponential model of Wykoff was the preferred model.

Apart from the effects of height-diameter realtionships on AGB, it is well recognizied that several other forest structural and taxonomic attributes affect AGB. With regard to forest structure, stem density and stand basal area, which tend to increase and decrease respectively with increasing altitude [36,42], affect AGB. With regard to taxonomic attributes, species richness has been linked with AGB. Generally, there is a decline in tree species richness with increasing altitude [36,43,44], because of a greater role of environmental filtering at higher elevations (e.g. cooler temperatures, fog, reduced light incidence and higher relative humidity). Environmental parameters, such as climate and soils also affect AGB [32,45,46]. Thus, quantifying the relationships of altitude on height-diameter relationships, stem density and stand basal area will assist in understanding how these contribute to AGB changes with temperature, an essential practical question, as almost all forests are currently warming, and will warm considerably in the future [47].

The Albertine rift is a biodiversity hotspot [48] with great forest structural and floristic diversity, and, therefore, is a conservation priority zone [49,50]. A carbon project could provide funds for enhacing conservation programmes in this region [51]. However, little is known about AGB estimates in the montane areas of the region. To our knowledge, no study has assessed AGB in tropical montane forests (TMF) in the Albertine Rift. In fact, few studies have assessed AGB in TMF in Africa and most have focused in Tanzania [52–56].

To improve our understanding of AGB estimates the factors affecting it, especially with regard to the Albertine Rift region, the objectives of this study were to: (1) determine how altitude and other factors affect height-diameter allometry in different TMFs, (2) assess how different height-diameter allometric models affect AGB estimates; and (3) determine how forest structural, taxonomic and environmental attributes affect AGB.

## Materials and methods

### Study area

**Fig 1:**
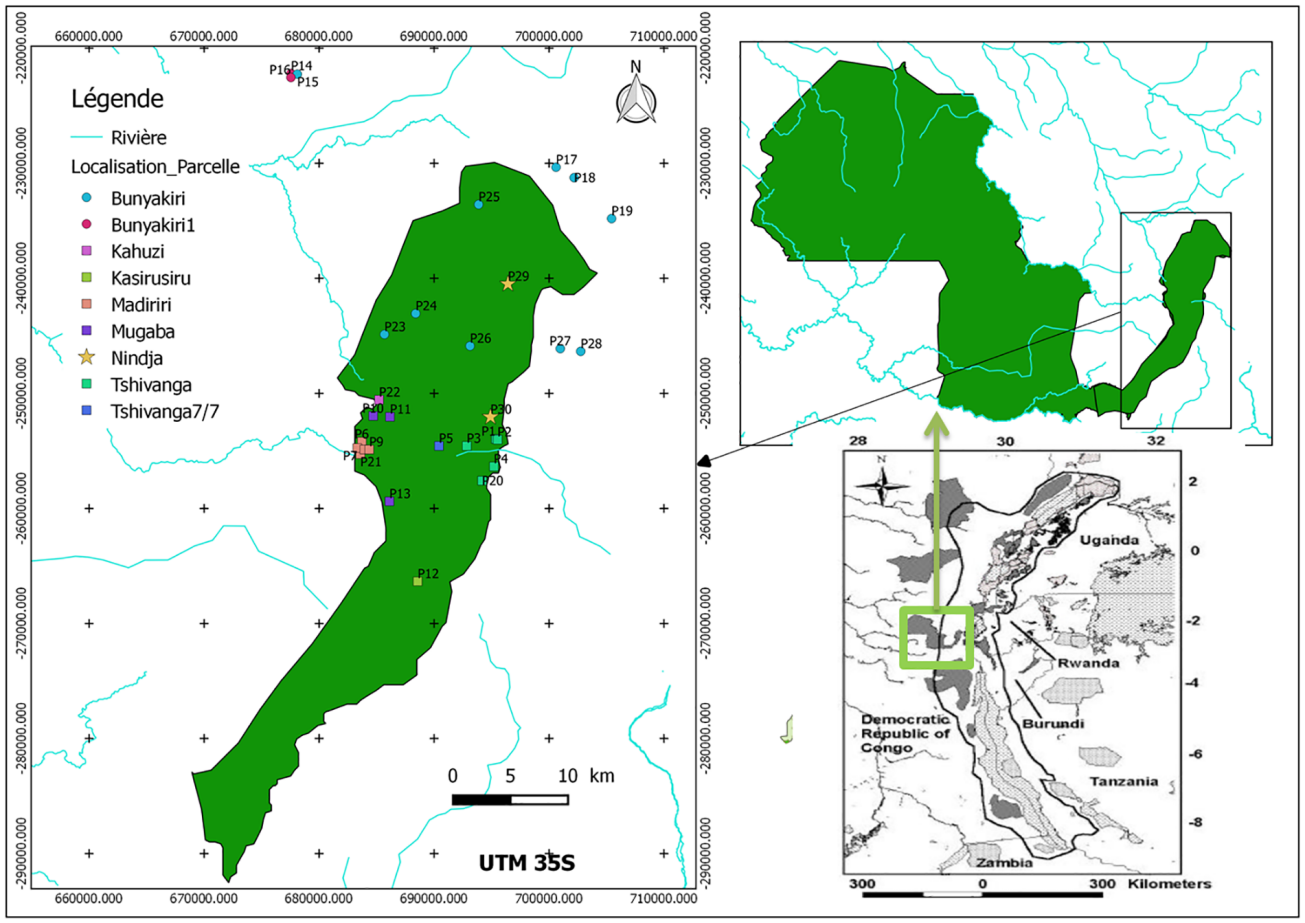
Study site and location of plots in and around Kahuzi Biega National Park. Background data adapted from Plumptre et al.[58].

This study focused on the montane forests of Kahuzi Biega National Park (NP) and its surroundings (Fig 1). A research permit was obtained from ICCN (Institut Congolais de Conservation de la Nature) and by the Director of Kahuzi-Biega National Park. To work in the community forest, permission was obtained from Mr Bulonvu, member of village committee. We confirm that the field studies did not involve endangered or protected species.

The Kahuzi Biega NP, located in eastern Democratic Republic of Congo (DRC), is part of the Albertine Rift region. Established as a forest reserve in 1937, it became a National Park of 6000km^2^ in 1975, with the aim of protecting the gorillas (*Gorilla beringei graueri*) found in the area [57]. In the NP altitude ranges from ~650 to ~3320m asl.

Kahuzi-Bienga NP has a bimodal rainfall regime, with most rains falling in September-December and March-April. Annual rainfall ranges between 1500 and 2000mm, mean annual temperature is 20°C, and humidity is close to 76% [59]. However, important climatic differences can be observed with increasing altitude (colder and wetter), with fog being a common feature above 2900m asl. Sometimes frost can be observed above 3300m asl, especially during the month of July. According to Dewitte et al. [60], Kahuzi-Bienga NP and its suroundings have a Acrisols and Ferrasols umbric soil types. The geological subtrate is volcanic rich in basalt [61].

Most of Kahuzi-Biega NP is covered by closed-canopy forest. While below 1250m asl there is semi-decidious and evergreen lowland rainforest, between 1250 and 2600m asl there are montane forests [59,62]. Montane forests are classified into four distinct types because of their floristic and structural characteristics: Sub montane (1250 to 1500m asl), lower montane (1500 to 1800m asl), middle montane (1800 to 2400m asl) and upper montane (2400 to 2600m asl) (see Imani et al. [44,63]). Sub montane forests are dominated by *Anonidium mannii*, *Strombosia scheffleri* and *Trichilia welwichii;* lower montane forests by *Trilepisium madagascariensis*, *Carapa grandiflora* and *Drypetes dinklagei*; middle montane forests by *Macaranga neomilbraediana*; and upper montane forests by *Hagenia abyssinica* and *Rapanea melanophloeos*. Sub montane and lower montane forests have higher floristic diversity and larger trees (diameter and height) than the other two forest types. Upper montane forests have shorter trees with twisted stems and many epiphytes on their branches. Natural bamboo (*Sinarundinaria alpine)* formations can be found in the upper montane forest.

The NP studied is part of the Albertine Afromontane Biodiversity Hotspot [48]. Several endangered animal and plants species are found in this NP, such as gorillas (*Gorilla beringei graueri*), elephant (*Loxodonta africana var*. *cyclotis*), forest buffalo (*Syncerus caffer nanus*), lion (*Panthera leo*) and giant forest hog (*Hylochoerus meinertzhageni*) [64,65]. Commercial logging never occurred on this NP. Surrounding communities, which are mainly farmers of Pygmy, Bashi and Lega tribes, are not allowed to hunt, fish, collect firewood or other non-timber forest products inside the NP, but some do. Because of civil unrest and lack of law reinforcement, some parts of the NP, mostly between 1400-1700m asl have been considerably degraded, due to firewood collection, mineral activities and charcoal production (Pers. Obs.).

### Study design and field measurements

**Fig 2:**
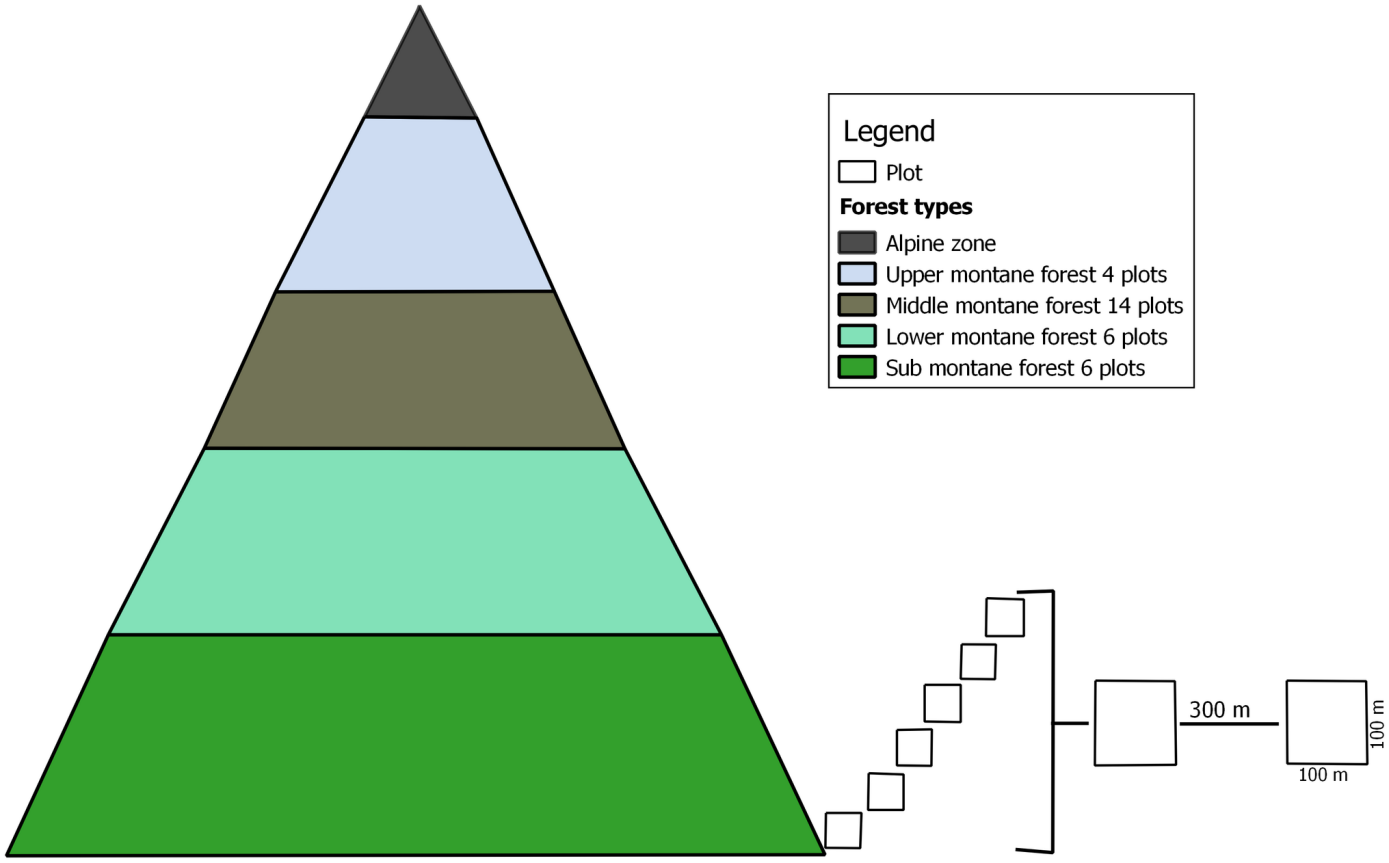
Montane forest stratification and sampling design.

Thirty plots of 1 ha were established at different altitudes within the montane forest belt. After preliminary observations, plots were set up randomly at least 300 m from each other in parts of the forest that were not degraded. Because of the abovementioned degradation between 1400-1700m asl inside the NP, twelve plots were established within this altitudinal zone in non-disturbed community forest outside the NP. After a cluster analysis, plots were grouped into four forest types in relation to their floristic and forest structure (see Imani et al. [44] for details), so that six plots were identified as sub montane forest, six as lower montane forest, 14 as middle montane forest and four as upper montane forest (Fig 2). Although a more balanced design would have been preferred we were unavailable to go back to the forest to set up more plots in e.g. upper montane forests. While the variation in forest structure in middle montane forests is greater than in other forest types (see Results), probably related to the plot being located in the windward/leeward side of the mountain, local variation in slope and exposure to wind, we are confident that taller trees or greater plot AGB would not be found even if more plots were set up in upper montane forests.

We assume that the forest plots studied have not been disturbed by direct human impacts, but may have been disturbed by natural events in the past.

In all plots all live free-standing woody stems ≥10 cm diameter at 1.3 m along the stem from the ground (or above buttresses/deformities if present) were measured and stems were identified to species when possible. Samples of unidentified trees were taken to the Herbarium of Centre for Research in Natural Sciences Lwiro (LWI), INERA Mulungu (MLGU) and deposited in Lwiro Herbarium. Unidentified trees represented 0.15% of the trees sampled (26 individuals of 16797 sampled), and they were found in Sub montane and lower montane forests. Taxonomy followed the plant list (www.theplantlist.org). Tree height was also measured in a number of trees per species and diameter class in each plot, following Arcangeli et al. [22]. In total, 3834 of the 16797 trees ≥10 cm diameter (23%) were sampled for height, using a hypsometer laser Ace 1000 Rangefinder. These included an average of 25% of trees in each plot covering a range of diameter classes. Plot altitude was recorded using a GARMIN GPSMAP 62 stc handheld Global Positioning System device.

In each plot four soil samples of the layer 0-30cm, which is the richest in organic matter [66], were collected. Each soil sample consisted of the combination of three cores collected within a subplot of 50 x 50m [67]. Soil samples for analysis were taken to IITA (International Institute of Tropical Agriculture) laboratory in Kalambo research station (DRC) where they were air-dried.

### Analysis of soil samples

Bulk density was determined using a Kopecky cylinder, from which soil was weighted and dried to constant weight [68]. Sand/silt/clay percentage was obtained using the hydrometer method [69] while soil pH, acidity, exchangeable Aluminium, Carbon, Nitrogen, Phosphorous and Potassium were determined using standard FAO protocols [69].

### The best height-diameter allometric model

Seven height-diameter allometric models commonly used to estimate height from diameter [21,25] were considered in this study: Gompertz or Winsor S-Shaped (Functions m1) [70], logistic (Function m2) [71], Weibull S-shaped (Function m3), Richards (Function m4), Michaelis–Menten (Model m5), Power (Model m6) and a second order polynomial (Model m7):

- Height = a*exp (−b * exp (−c*diameter) (m1)
- Height = a/(1 + exp[(b − diameter) / c] (m2)
- Height=a+(b−a)exp[−exp(c)diameter ^d] (m3)
- Height = a+(b−a) exp[−exp(c)*diameter] (m4)
- Height = a*_diameter/(b+diameter) (m5)
- $$\text{Height}\;=\;\;a*\_diameter^{b}$$ (m6)
- $$\text{Height}\;=\;a+b*log(diameter)+c*{(log(diameter))}^{2}$$ (m7)

Where a, b, c and d are model parameters estimated using the nls function in nlme and minpack.lm packages of R [72]. The best model for (i) each plot and (ii) each forest type (sub, lower, middle and upper montane) were assessed. The best model was selected considering: Akaike Information Criterion or AIC [73], Root Mean Squared Error (RMSE) and variation explained by the model (R^2^). Overall, the best model is one which has low AIC and RMSE but high R^2^ [21,73,74].

### Factors affecting the slenderness coefficient

The slenderness coefficient (the mean of the ratio between height and diameter of all individuals measured on field) was calculated for each plot [75]. Fifteen variables, including altitude, slope, stem density, tree species richness, and eleven soil variables (pH, H+, exchangeable Aluminium (Al^3+^), Carbon, Nitrogen, Phosphorous, bulk density, sand, silt, and clay percentage) were assessed as potential factors affecting the slenderness coefficient. Mean plot slope was extracted from ASTER global digital elevation model maps (https://wist.echo.nasa.gov/~wist/api/imswelcome/) at 30m pixel resolution using the slope function in ArcGIS. Stem density (number trees ha^-1^) included all trees ≥10 cm diameter. Tree species richness was determined as total number of tree species (identified species with a valid Latin name plus unique morphospecies) ≥10 cm diameter observed in a given plot.

### AGB estimates

The Chave et al. [30] equation including diameter, wood density and tree height was used to estimate the AGB of each tree in the plot. The best taxonomic match of wood density to each stem was extracted from a global database [76,77] following Lewis et al. [32]. For the trees whose height was not sampled in the field, their height was estimated using (1) the best height-diameter allometric model for each plot as determined in previous section, (2) the best height-diameter allometric model for each forest type as determined in previous section, (3) the Feldpausch et al. [17] height-diameter allometric model for East Africa and (4) the Feldpausch et al. [17] height-diameter allometric model for Central Africa.

### Statistical analysis

R statistical software R 3.02 was used for all statistical analyses [78]. Anova and Tukey-HSD tests or Kruskal-Wallis (if non homogeneity of variance, for H_mean_ and D_mean_) and multiple comparison of Kruskal-Wallis, using ‘kruskalmc’ in R, were used to evaluate significant differences in (i) soil characteristics, (ii) structural parameters (mean height, mean diameter, basal area, stem density, stem density of large tree ≥ 50cm, woody mass density, species richness, AGB) and (iii) slenderness coefficients between forest types, after data transformation (orthogonality).

A linear regression and a pearson correlation test were used to (i) assess how altitude affected height-diameter relationships and (ii) which environmental attributes are correlated to slenderness coefficient. A multiple regression model was used to investigate how environmental attributes affected height-diameter relationships. In this later case only variables with p<0.05 were retained in the final model using ‘update function’. Before running this regression, a correlation between soil variables was assessed using Pearson correlation. Only non-auto correlated variables (significant at p< 0.01) were considered in the multiple regression. Thereby, the variables retained in this analysis were Al^3+^, K^+^, P, C/N ratio, silt, sand, bulk density, slope and stem density. Variables were randomly permuted in the multiple regression, so that variable order did not affect the p value.

AIC, RMSE and R^2^ were used to determine the best height-diameter model for each forest type. When AIC was similar between two models, a student test was applied to confirm if the tree heights estimated using one or another model were significantly different from each other. Pearson correlation was used to assess the relationship between structural parameters, soil characteristics and AGB. T-tests were used to determine differences between AGB calculated using different approaches presented in the previous section.

## Results

### Height-diameter allometric models

According to AIC, and RSME values, the model that performed better was not the same across forest types and mountains (Table 1). The Gompertz (Model m1) performed better for Sub montane and lower montane forests while the Richards Asymptotic (Model m4) performed better for the middle montane forests and the second order polynomial (Model m7) performed better for the upper montane forests (Table 1).

**Table 1:** Local site specific height-diameter allometric models relating height (in m) to diameter (in cm).

Forest type	Models	*Models parameters*				*Selection criteria*	
		a	b	c	d	RSME	AIC
Sub montane (1250–1500)	**Model 1**	**30.61**	**2.7**	**0.95**		**3.66**	**4698.36**
	Model 2	29.2	26.8	0.71		3.68	4704.6
	Model 3	30.3	27.8	-5.5	1.5	3.66	4699.18
	Model 4	34	-3.1	-3.7		3.69	4709.55
	Model 5	60.5	81.98			3.8	4757.88
	Model6	1.50	0.69			4;03	4862.17
	Model7	-12.31	5.27	0.96		3.76	4741.57
Lower montane (1500–1800)	**Model 1**	**30.0**	**3.2**	**0.94**		**3.07**	**4983.82**
	Model 2	28.04	24	10.3		3.09	4993.32
	Model 3	29.04	27.2	-5.8	1.7	3.08	4988.48
	Model 4	33.4	-5.2	-3.5		3.12	5014.14
	Model 5	70.25	97.11			3.34	5146.39
	Model6	1.24	0.75			3.58	5281.67
	Model7	-15.02	6.45	0.91		3.2	5065.38
Middle montane (1800–2400)	Model 1	21.54	2.3	0.94		2.98	8201.36
	Model 2	20.88	19	11.5		3.01	8228.07
	Model 3	23.29	27	-2.8	0.9	2.97	8186.18
	**Model 4**	**22.7**	**-1.71**	**-3.3**		**2.97**	**8184.93**
	Model 5	33.89	40.85			3.02	8234.56
	Model6	2.09	0.55			3.22	8453.27
	Model7	-18.68	12.08	-0.65		2.98	8199.96
Upper montane (2400–2600)	Model 1	12	2.3	0.9		2.42	1672.03
	Model 2	11.8	11.3	7.4		2.43	1673.65
	Model 3	na	na	na	na	na	na
	Model 4	12.3	-2.8	-2.6		2.42	1670.35
	Model 5	17.21	19.65			2.43	1672.65
	Model6	2.34	0.43			2.49	1688.49
	**Model7**	**-15.26**	**11.57**	**-1.17**		**2.41**	**1667.91**

The performed model was selected using Akaike Information Criteria (AIC) and Root Mean Squared Error (RSME). The best model for each forest type is shown in bold.

If each plot is considered separately, there is limited agreement between plots of the same forest type. The Logis (Model m2) performed better for 50% of the plots in both the Sub montane and lower montane forests, and for 35% of the middle montane forests. The Power (Model m6) performed better for 50% of the upper montane forests (see S1 Table). For the middle montane forests, for which 14 plots were sampled, four different models were preferred depending on the plot, highlighting the variability in height-diameter allometry even within a given forest type. The Michaelis-Menten (Model m5) and the Weibull (Model m3) only outperformed other models in four and one plots respectively of the 30 plots sampled (S1 Table).

### The effects of height-diameter allometric model choice on AGB estimates

**Fig 3:**
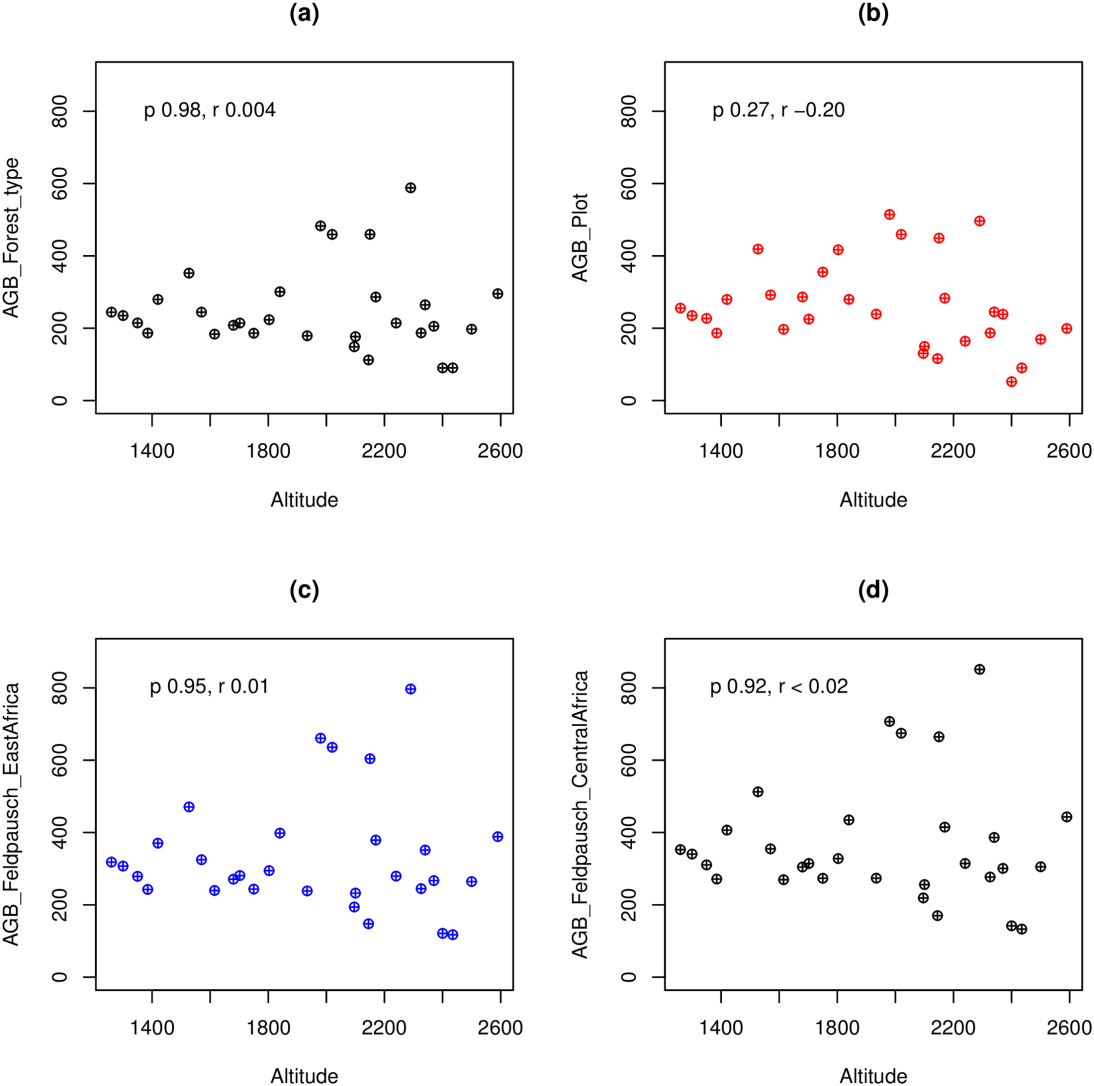
Above ground biomass (AGB in Mg ha-1) with regard to altitude. AGB calculated using (a) the best height-diameter model per forest type, (b) the best height-diameter allometric model per plot, (c) Feldpausch et al. (2012) height-diameter allometric model for East Africa and (d) Feldpausch et al. (2012) height-diameter allometric model for Central Africa. p value and “r” of Pearson correlation between AGB and altitude are indicated in each plot. Significant correlations at p<0.01.

The use of different height-diameter allometric models had important effects on AGB estimates (Fig 3, Table 2). Compared with using the best height-diameter allometric model per plot, the use of the best model per forest type had little effect on AGB estimates in Sub montane and middle montane forests (-4.3 and 3.7% change in AGB respectively), but considerable effects in lower and upper montane forests (-24.9 and 34.67% change in AGB respectively, see Table 2). However, these differences were not significant if forest type is not taken into account (Table 2).

**Table 2:** Impact of model choice in above ground biomass (AGB in Mg ha^-1^) estimation.

Forest type	AGB (a)	AGB (b)	AGB (c)	AGB (d)	% change (method a-b)	% change (method a-c)	% change (method a-d)
Sub montane	267.1 ± 80.5	252 ± 58.1	331.3 ± 80.4	365.6 ± 84.9	(-) 4.3 ± 6.2	25.5 ± 7.4	38.8 ± 8.9
Lower montane	295.3 ± 81.3	209.9 ± 23.1	275.7 ± 32.1	307.4 ± 32.6	(-) 24.9 ± 18.9	(-)1.4 ± 24.8	10 ± 27.6
Middle montane	282.2 ± 140.2	290.3 ± 147.9	387.8 ± 203.8	424.5 ± 213.2	3.7 ± 14.2	37.6 ± 18.5	52.1 ± 19.7
Upper montane	127.7 ± 68.2	168.3 ± 98.6	222.7 ± 129.9	255.8 ± 147.9	34.6 ± 32.7	78.4 ± 45	105.7 ± 54.4
Significance t test					p = 0.61	p<0.001	p<0.001

AGB was calculated using (a) the best height-diameter allometric model per plot, (b) the best height-diameter model per forest type, (c) Feldpausch et al. (2012) height-diameter allometric model for East Africa and (d) Feldpausch et al. (2012) height-diameter allometric model for Central Africa; and the relative change in AGB (in %) between the different methods used.

The use of Feldspauch East Africa height-diameter allometric model produced even greater overestimates: 25.5%, 37.6% and 78.4% change in AGB in Sub montane, middle and upper montane forests respectively (Table 2).

The use of Feldspauch Central Africa height-diameter allometric model also produced great overestimates: 38.8%, 52.1% and 105% in Sub montane, middle and upper montane forests respectively (Table 2). For lower montane forests, Feldspauch East Africa and Feldspauch Central Africa models produced smaller changes on AGB (-1.4 and 10%, see Table 2). These differences were all significant even if forest type was not taken into account (Table 2).

### AGB estimates and its relationship with forest and environmental attributes

AGB ranged from 168 Mg ha^-1^ in the upper montane forests to 290 Mg ha^-1^ in middle montane forests (Table 3). However, no significant differences in AGB were observed among the forest types studied (Table 3). This is related to the fact that upper montane forests had significantly lower mean height, mean diameter, and number of species but greater stem density than other forest types (Table 3). If individual plots are considered, AGB ranged from 90 Mg ha^-1^ in one plot in the upper montane forest to 588 Mg ha^-1^ in a plot in middle montane forests (see Fig 3). If AGB is plotted against altitude, AGB has a certain bell-shape relationship with altitude (see Fig 3).

**Table 3:** Structural and environmental attributes per forest type.

Forest types	Hmean		Dmean		BA		SD		SD50		WMD		No spp		AGB	
Sub montane	13 ± 0.6	a	27 ± 1.3	a	35.6 ± 4.5	a	438.3 ± 24.5	a	15.2 ± 6.6	a	0.6 ± 0.03	a	53.3 ± 8.9	a	252 ± 58.1	a
Lower montane	11.7 ± 0.3	ab	23.3 ± 0.8	ab	30.5 ± 1.7	a	511.2 ± 54.4	a	10.5 ± 2.1	a	0.6 ± 0.02	a	53.7 ± 5.8	a	209.9 ± 23.1	a
Middle montane	12 ± 1.4	ab	25.2 ± 5.3	ab	38.6 ± 13.4	a	546.4 ± 200.7	a	10.5 ± 6.4	a	0.6 ± 0.04	a	27.1 ± 10.5	b	290.3 ± 147.9	a
Upper montane	10.2 ± 0.6	b	18.9 ± 1.7	b	29.3 ± 16.3	a	869.3 ± 477.8	b	6.8 ± 7.6	a	0.6 ± 0.03	a	13.8 ± 5.6	c	168.3 ± 98.6	a

Mean height (mean height of all trees in the plot, H_mean_), mean diameter (mean diameter of all trees in the plot, D_mean_), basal area (BA in m^2^ ha^-1^), stem density (SD in number stems ha^-1^), stem density of large trees (with diameter >50cm, SD_50_ in number stems ha^-1^), wood mass density (WMD), species richness (No spp) and above ground biomass (AGB in Mg ha^-1^), per forest type. Different letters within columns mark significant differences at p<0.05.

AGB was found to be significantly positively correlated with BA, SD_50_, D_mean_ and H_mean_ but not with stem density, WMD or species richness (Table 4, S1 Fig). With regard to environmental attributes, AGB was significantly negatively correlated with soil pH (at p < 0.05) and significantly positively correlated with potassium (Table 4, S1 Fig).

**Table 4:** Correlation between above ground biomass (AGB in Mg ha^-1^) and different forest attributes.

	AGB	
BA	0.94	**
SD50	0.62	**
Dmean	0.79	**
Hmean	0.64	**
SD	-0.17	
WMD	0.27	
No spp	0.04	
pH	-0.35	*
H+	-0.01	
Al	0.15	
K	0.52	**
P	0.17	
C	-0.22	
N	-0.16	
C/N	-0.08	
Clay	-0.12	
Sand	0.14	
Silt	-0.02	
Bulk Density	0.17	
CEC	-0.19	
Slope	0.07	
Altitude	-0.001	

Forest attributes including mean height (mean height of all trees in the plot, H_mean_), mean diameter (mean diameter of all trees in the plot, D_mean_), basal area (BA in m2 ha-1), stem density (SD in number stems ha-1), stem density of large trees (with diameter >50cm, SD_50_ in number stems ha-1), wood mass density (WMD_)_, species richness (No spp), Altitude (Alt, meter) and several soil characteristics and slope.

Significant correlations:

** p<0.01

* p<0.05

### Slenderness coefficients and soil samples

**Fig 4:**
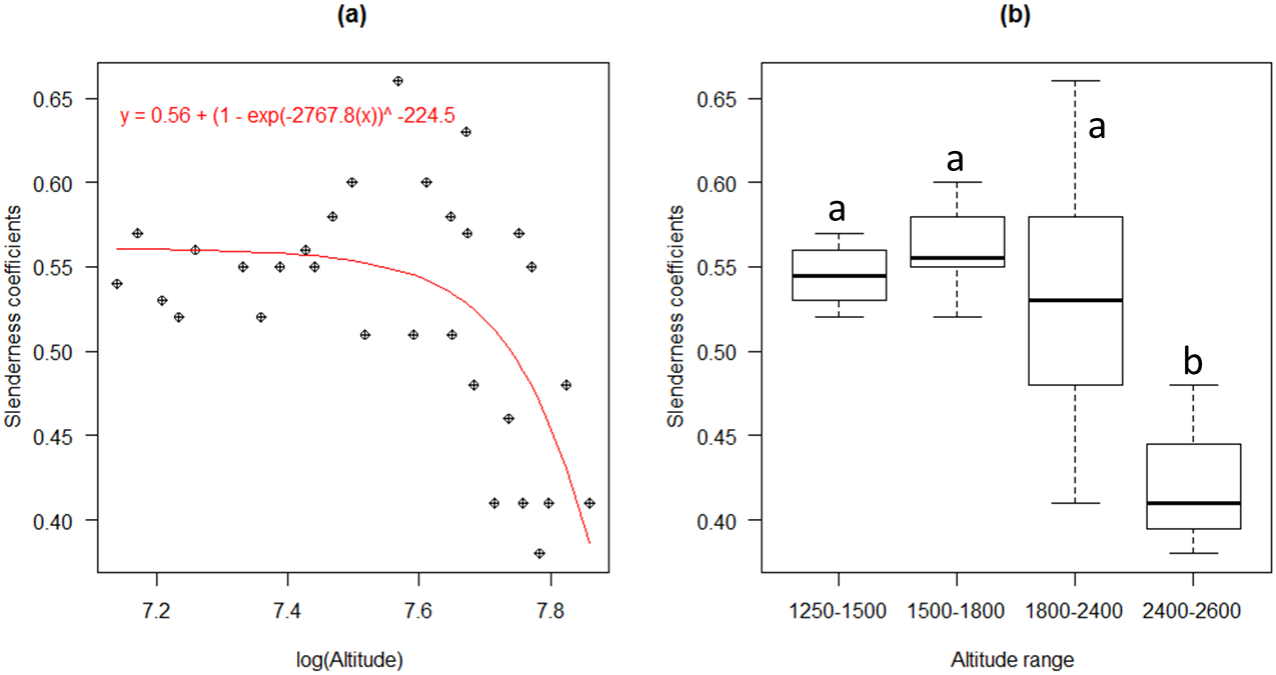
Slenderness coefficients with regard to altitude (a) and forest type (b). Different letters in figure b indicate significant differences at p<0.05.

Slenderness coefficients significantly changed with increasing altitude (R^2^ = 0.39; r– 0.474, p<0.01, see Fig 4a). Upper montane forest had a significantly different slenderness coefficient compared with the other forest types (Fig 4b).

Among the forest and environmental attributes considered, potassium content, sand and silt content significantly affected the slenderness coefficient (Table 5). Many soil variables were auto correlated (S2 Table).

**Table 5:** Forest and environmental attributes which significantly affected slenderness coefficients in montane forests of Kahuzi Biega NP.

Parameters	Coefficient	Standard Error	p
Potassium (K+)	0.53	0.25	0.04**
Sand	-0.003	0.001	0.007***
Silt	-0.004	0.001	0.003***

(R-squared of the linear regression = 0.354 p = 0.009).

Signif. codes:

*** 0.001

** 0.01

With regard to soil characteristics, important differences were observed between forest types (S3 Table). In general, Sub montane forests and upper montane forests were the most different, with lower and middle montane forests having values ranging between both extremes. Soil characteristics are discussed in detail in another publication (Imani et al. in preparation [79]).

## Discussion

### Height-diameter allometric models

Banin et al. [35] showed that lowland tropical forests on different continents have differing height-diameter allometry. Within continents, different regions have differing height-diameter allometry [17,18]. At a smaller scale Fayolle et al. [25] demonstrated that different height-diameter allometric models were needed for the two types of lowland rainforest they studied (evergreen and semi-deciduous). Our results show that different height-diameter allometric models should be used depending upon forest types in montane areas—continent or regional models being potentially biased. Moreover, our results also indicate that there is considerable variation within forest types at the plot level, and different height-diameter allometric equations might be preferred even within one forest type. This finding might suggest that individual models for respective tree species could perform best. However, building height-diameter allometric models for different species is often challenging as a great number of stems per size-class is required. A recent study form Australia showed that using generic allometric models based on plant functional types rather than species-specific models [80]. Moreover, in montane forests, the same species can have different heights for a given diameter depending on exposure to wind, windward/leeward side of mountain, slope or other factors (soil) (see [81]). Therefore, we suggest the use of ‘forest type’ or ‘plot level’ approach when building height-diameter allometric models (see further Discussion).

Interestingly, the Michaelis-Menten model, highlighted as the best in two recent studies in lowland rainforests [25,33] was not the ideal in our study area. The Weibull model, also commonly preferred for lowland rainforests (e.g. [17]) and in montane forests in Peru [26] was not the preferred in our study area neither. Our results do not support the notion that a truly asymptotic model, more realistic biologically, is preferred over other types of height-diameter allometric models [23,31]. For instance, the second order polynomial model outperformed asymptotic models in upper montane forests, which tends to have much shorter trees.

Most studies choose the same model for the different forest types they study (eg., [19,33]), and discuss the different values of the parameters in the selected model. Here we highlight the fact that considering the great structural variation in TMFs, different models might be preferred, and this should be first investigated.

### AGB per forest type

Despite the significant differences in forest structure and tree species richness [63], no significant differences in AGB were observed among forest types. Forests at higher altitude had smaller trees (height and diameter) but greater stem density. Although no significant differences between forest types were observed, AGB was found to have a certain relationship with altitude close to a bell-shape. Other studies in TMFs have reported that AGB decreased with increasing altitude (e.g.,[36,82]) or had a bell-shaped relationship with altitude as lower sites were drier (e.g. [53,55]). It should be noted that in our study, lower sites are not drier, and that we did not assess lowland rainforest type.

In our study mean AGB per forest type ranged from 168 to 290 Mg ha^-1^. These values are within those reported by Spracklen and Righelato [83] for the world’s tropical montane forests (77–785 Mg ha^-1^). However, we expected higher biomass in middle montane forests (only four plots of the middle montane forest had >300 Mg ha^-1^). It has been reported that Asian and Neotropical TMFs have similar mean AGB (257 and 247 Mg ha^−1^, n = 31 and 56, respectively) while that of African TMFs is higher (527 Mg ha^−1^ n = 7) [83]. Other studies support this finding: Ensslin et al. [55] emphasised the high AGB found in *Podocarpus* dominated forest on Mt Kilimanjaro in Tanzania (364 Mg ha^−1^). *Podocarpus*, *Ocotea usambariensis* or *Faurea saligna* (the species which largest individuals linked with high biomass in those forests) are not abundant in our study area, which could explain these differences (for more details see, [44,63]).

### The effects of forest attributes and environment on AGB

Several authors have shown that average tree diameter, large tree density and stand basal area tend to be better predictors of AGB than overall tree density [32,46,84]. This was also the case in our study area. Interestingly, we did not find any significant relationship between AGB and tree species richness in our 1-ha plots. Poorter et al. [46] showed that there was a consistent significant positive relationship between AGB and taxonomic attributes at the 0.1-ha scale, whereas this relationship disappeared at the 1-ha scale (study focused on the Amazon lowland rainforest). Chisholm et al. [85] also found that diversity–biomass relationships were strong and positive at very small spatial scales (20 m × 20 m), whereas at larger spatial scales (0.25 and 1 ha) there was no consistent relationship. A recent study by Sullivan et al. [86] also showed that there is no relationship between AGB and taxonomic attributes at 1-ha scale in African lowland tropical forests.

With regard to environmental parameters, AGB was found to be positively correlated with soil potassium but negatively correlated with soil pH. With regard to soil potassium, our observations could be related to increased cation exchange capacity and higher turnover rates at sites with higher fertility soils ([87]). Several studies have shown that rainforest areas with higher values of soil K concentration support greater AGB (e.g., [88] for lowland rainforest, [89] for TMFs in the Equatorial Andes, [90] in Amazonian forest). A long-term fertilization experiment in lowland rainforest demonstrated that K addition increased stand-level biomass and production of fine root biomass, enhanced seedling tissue nutrient concentrations, reduced seedling root allocation and improved stomatal control and photosynthesis [91].

While the positive correlation of K with AGB was expected, the negative relationship with soil pH was rather unexpected. In general, soil fertility declines with elevation, related to soil water logging, elevated soil acidity in combination with putative Al^3+^ toxicity, shortage of N or other nutrients, recalcitrant litter with slow decomposition, and others [92]. This soil acidity is especially related to slow litter decomposition, plant and microorganisms activities [92,93]. It has also been highlighted that low fertility soils, apart from slower growth, also have slower turnover (lower mortality, longer carbon residence times) [32,90]. However, Fujii [93] reported that plant productivity can be high on some acidic soils; and no relationship between soil pH and AGB was found in the Equatorial Andes [89]. Other nutrient-cycling mechanisms apart from direct nutrient absorption from soil (e.g. the nutrient uptake from litter, or the storage of nutrients in the biomass) also affect forest biomass (see [94]), but have been understudied (we did not assess them either). Overall, we can conclude that forest structure had a greater effect on AGB than tree diversity. Soil attributes also significantly affected AGB, but more research is needed to disentangle the effects of soil characteristics on AGB in TMFs.

### The importance of selecting a local height-diameter allometric model for AGB estimates

Several authors have shown the significant biases in AGB estimates associated with using regional height-diameter models [19,23–26] and have highlighted the need for local site-specific models. Our results show that using a local height-diameter allometric model for AGB estimates significantly reduces overestimation of AGB compared with Feldpausch et al. [17] model for East or Central Africa (up to 78 and 105% overestimates respectively). Moreover, our results also highlight that using a height-diameter allometric model per plot is even better, because of the considerable variation in forest structure and slenderness coefficients within one forest type in TMFs. To our knowledge, this is the first study which highlights this. As accurately determining AGB stocks in tropical forests is a major interest for REDD+ and climate mitigation mechanisms [95], this should be considered in future studies on TMFs.

## Supporting information

S1 Table Plot specific height-diameter allometric models relating height (in m) to diameter (in cm), the Akaike Information Criteria (AIC), variation explained by the model (R^2^) and the Root Mean Squared Error (RSME). (DOCX)

S2 Table Correlation between soils attributes. (DOCX)

S3 Table Soil main characteristics per forest type. (DOCX)

S1 Fig Correlation between above ground biomass (AGB in Mg ha^-1^) and others floristics, soil and structural attributes. (TIF)
