# Supplementary material for: Height-diameter allometry and above ground biomass in tropical montane forests: Insights from the Albertine Rift in Africa
Source: PLoS One. 2017 Jun 15;12(6):e0179653. doi: 10.1371/journal.pone.0179653 (PMC5472301; doi:10.1371/journal.pone.0179653)
Supplement: S1 Table — (DOCX) [file pone.0179653.s001.docx]

**S1 Table.**  Plot specific height-diameter allometric models relating height (in m) to diameter (in cm), the Akaike Information Criteria (AIC), variation explained by the model (R²) and the Root Mean Squared Error (RSME). The best model for each plot is shown in bold.

| **Forest type** | **Plot** | | **Gompertz (m1)** | | | | **Logis (m2)** | | | | | **Weibull (m3)** | | | | | | | **Richards Asymptotic (m4)** | | | | |
| --- | --- | --- | --- | --- | --- | --- | --- | --- | --- | --- | --- | --- | --- | --- | --- | --- | --- | --- | --- | --- | --- | --- | --- |
|  |  |  | **AIC** | **R²** | **RSME** | | **AIC** | | **R²** | | **RSME** | **AIC** | | **R²** | | **RSME** | | | **AIC** | | **R²** | | **RSME** |
| Sub montane | **P23** | | 692,4 | 0,8 | 3,43 | | 695,9 | | 0,8 | | 3,48 | na | | na | | na | | | **689,2** | | **0,81** | | **3,39** |
|  | **P24** | | 868,3 | 0,79 | 4,3 | | 871,3 | | 0,78 | | 4,3 | 869,2 | | 0,79 | | 4,24 | | | **867,9** | | **0,79** | | **4,25** |
|  | **P25** | | 1049,16 | 0,794 | 3,97 | | **1047,4** | | **0,796** | | **3,96** | na | | na | | na | | | na | | na | | na |
|  | **P26** | | 676,84 | 0,802 | 3,3 | | **675,45** | | **0,804** | | **3,28** | 677,6 | | 0,804 | | 3,28 | | | 681,08 | | 0,795 | | 3,35 |
|  | **P27** | | 669,46 | 0,815 | 2,41 | | 675,29 | | 0,807 | | 2,45 | na | | na | | na | | | **662,24** | | **0,822** | | **2,36** |
|  | **P28** | | 663,04 | 0,717 | 3,26 | | **662,13** | | **0,719** | | **3,24** | 663,89 | | 0,72 | | 3,24 | | | 666,62 | | 0,709 | | 3,3 |
| Lower montane | **P14** | | 635,46 | 0,812 | 2,98 | | **633,35** | | **0,815** | | **2,95** | 635,23 | | 0,815 | | 2,95 | | | 641,37 | | 0,802 | | 3,05 |
|  | **P15** | | **679,05** | **0,875** | **3,02** | | 681,33 | | 0,873 | | 3,04 | 681,21 | | 0,875 | | 3,02 | | | 684,82 | | 0,869 | | 3,08 |
|  | **P16** | | **759,13** | **0,885** | **2,61** | | 764,3 | | 0,881 | | 2,65 | 761,42 | | 0,885 | | 2,61 | | | 765,51 | | 0,881 | | 2,66 |
|  | **P17** | | 781,58 | 0,893 | 2,52 | | **780,81** | | **0,894** | | **2,52** | 782,32 | | 0,894 | | 2,51 | | | 792,79 | | 0,886 | | 2,61 |
|  | **P18** | | 1219,36 | 0,795 | 3,02 | | **1217,96** | | **0,796** | | **3,01** | 1220,1 | | 0,796 | | 3,01 | | | 1228,6 | | 0,787 | | 3,08 |
|  | **P19** | | 810,38 | 0,816 | 3,07 | | 804,85 | | 0,822 | | 3,01 | **804,48** | | **0,825** | | **2,99** | | | 822,05 | | 0,802 | | 3,18 |
| Middle montane | **P1** | | 297,05 | 0,612 | 2,69 | | 297,7 | | 0,608 | | 2,71 | na | | na | | na | | | 296,3 | | 0,617 | | 2,67 |
|  | **P2** | | 363,41 | 0,768 | 1,82 | | **363,37** | | **0,768** | | **1,82** | 365,31 | | 0,768 | | 1,82 | | | 364,04 | | 0,766 | | 1,83 |
|  | **P3** | | 339,85 | 0,687 | 3,23 | | **338,95** | | **0,691** | | **3,21** | na | | na | | na | | | na | | na | | na |
|  | **P4** | | 708,13 | 0,757 | 2,35 | | 709,81 | | 0,755 | | 2,36 | 708,43 | | 0,76 | | 2,34 | | | **706,71** | | **0,759** | | **2,34** |
|  | **P5** | | **346,1** | **0,799** | **2,17** | | 346,49 | | 0,798 | | 2,18 | 348,16 | | 0,799 | | 2,17 | | | 346,63 | | 0,798 | | 2,18 |
|  | **P6** | | **883,74** | **0,688** | **2,23** | | 884,04 | | 0,687 | | 2,24 | na | | na | | na | | | na | | na | | na |
|  | **P7** | | 583,43 | 0,811 | 2,04 | | 587,8 | | 0,805 | | 2,07 | 581,27 | | 0,817 | | 2,01 | | | **580,14** | | **0,816** | | **2,01** |
|  | **P8** | | **892,71** | **0,788** | **3,17** | | 894,51 | | 0,785 | | 3,18 | 894,43 | | 0,788 | | 3,16 | | | 893,64 | | 0,786 | | 3,18 |
|  | **P9** | | 826,65 | 0,753 | 2,77 | | 829,97 | | 0,748 | | 2,79 | 825,22 | | 0,759 | | 2,74 | | | 824,12 | | 0,757 | | 2,75 |
|  | **P20** | | 514,05 | 0,795 | 2,18 | | **512,99** | | **0,797** | | **2,17** | na | | na | | na | | | 519,39 | | 0,785 | | 2,24 |
|  | **P21** | | 917,96 | 0,707 | 3,41 | | 925,59 | | 0,694 | | 3,49 | na | | na | | na | | | 910,63 | | 0,719 | | 3,34 |
|  | **P22** | | 448,05 | 0,804 | 3,03 | | **446,94** | | **0,806** | | **3,02** | 449,14 | | 0,806 | | 3,02 | | | 450,88 | | 0,797 | | 3,08 |
|  | **P29** | | 454,06 | 0,679 | 3,34 | | **453,76** | | **0,677** | | **3,33** | 455,77 | | 0,677 | | 3,33 | | | 455,37 | | 0,671 | | 3,36 |
|  | **P30** | | 267,75 | 0,747 | 2,36 | | 270,47 | | 0,735 | | 2,42 | na | | na | | na | | | 264,39 | | 0,762 | | 2,29 |
| Upper montane | **P10** | | na | na | na | | na | | na | | na | na | | na | | na | | | na | | na | | na |
|  | **P11** | | na | na | na | | na | | na | | na | na | | na | | na | | | na | | na | | na |
|  | **P12** | | 225,78 | 0,609 | 1,75 | | 226,2 | | 0,606 | | 1,76 | na | | na | | na | | | **225,18** | | **0,613** | | **1,74** |
|  | **P13** | | 800,82 | 0,523 | 2,11 | | **800,59** | | **0,524** | | **2,11** | na | | na | | na | | | 801,39 | | 0,522 | | 2,11 |
|  |  | | **a** | **b** | **c** | | **a** | | **b** | | **c** | **a** | | **b** | | **c** | | **d** | **a** | | **b** | | **c** |
| Sub montane | **P23** | | 32,50 | 2,60 | 0,96 | | 30,40 | | 29,40 | | 15,00 | na | | na | | na | | na | 39,24 | | **-2,10** | | -3,95 |
|  | **P24** | | 31,60 | 2,90 | 0,95 | | 30,70 | | 26,20 | | 12,40 | 32,10 | | 33,40 | | -4,50 | | 1,28 | 33,40 | | -6,60 | | -3,40 |
|  | **P25** | | 32,90 | 3,02 | 0,95 | | 31,57 | | 28,19 | | 12,43 | na | | na | | na | | na | na | | na | | na |
|  | **P26** | | 26,57 | 2,73 | 0,94 | | 25,66 | | 23,59 | | 11,67 | 25,51 | | 21,59 | | -6,65 | | 1,91 | 28,50 | | -3,72 | | -3,41 |
|  | **P27** | | 32,14 | 2,09 | 0,97 | | 29,06 | | 28,18 | | 17,57 | na | | na | | na | | na | 40,23 | | 1,59 | | **-4,20** |
|  | **P28** | | 27,29 | 2,53 | 0,95 | | 26,16 | | 24,41 | | 12,99 | 25,88 | | 21,19 | | -6,80 | | 1,92 | 30,05 | | **-2,37** | | -3,58 |
| Lower montane | **P14** | | 24,69 | 3,25 | 0,93 | | 23,82 | | 20,51 | | 9,01 | 23,29 | | 19,20 | | -7,67 | | 2,32 | 26,44 | | -6,75 | | -3,15 |
|  | **P15** | | 33,98 | 3,08 | 0,95 | | 31,99 | | 25,48 | | 11,22 | 33,33 | | 31,11 | | -5,88 | | 1,66 | 38,99 | | -5,24 | | -3,61 |
|  | **P16** | | 33,26 | 3,36 | 0,94 | | 31,28 | | 25,19 | | 10,27 | 32,91 | | 31,78 | | -5,75 | | 1,64 | 39,45 | | -5,81 | | -3,64 |
|  | **P17** | | 33,51 | 2,92 | 0,95 | | 32,21 | | 29,85 | | 13,03 | 32,44 | | 29,05 | | -6,74 | | 1,83 | 38,09 | | -2,94 | | -3,85 |
|  | **P18** | | 25,62 | 3,16 | 0,93 | | 24,40 | | 21,09 | | 9,29 | 24,46 | | 21,43 | | -6,59 | | 1,97 | 28,27 | | -5,91 | | -3,30 |
|  | **P19** | | 26,98 | 3,78 | 0,92 | | 25,92 | | 21,02 | | 7,94 | 25,05 | | 20,15 | | -10,35 | | 3,16 | 29,99 | | -7,40 | | -3,26 |
| Middle montane | **P1** | | 23,69 | 1,76 | 0,95 | | 22,43 | | 17,79 | | 14,25 | na | | na | | na | | na | 26,75 | | 1,94 | | -3,61 |
|  | **P2** | | 17,45 | 1,85 | 0,92 | | 17,19 | | 12,34 | | 10,01 | 17,27 | | 13,51 | | -4,49 | | 1,46 | 17,80 | | **-9,92** | | -2,84 |
|  | **P3** | | 22,41 | 1,52 | 0,96 | | 22,19 | | 21,02 | | 20,34 | na | | na | | na | | na | na | | na | | na |
|  | **P4** | | 19,26 | 3,02 | 0,91 | | 18,70 | | 15,56 | | 7,49 | 23,12 | | **46,45** | | **-1,33** | | **0,57** | 20,40 | | -5,77 | | -2,86 |
|  | **P5** | | 19,96 | 2,16 | 0,93 | | 19,51 | | 16,21 | | 10,69 | 19,85 | | 17,32 | | -4,52 | | 1,41 | 20,79 | | -1,94 | | -3,08 |
|  | **P6** | | 18,69 | 2,63 | 0,91 | | 18,33 | | 13,21 | | 7,51 | na | | na | | na | | na | na | | na | | na |
|  | **P7** | | 20,75 | 1,99 | 0,94 | | 20,35 | | 16,45 | | 11,56 | 22,30 | | 31,60 | | -1,89 | | 0,68 | 21,34 | | -2,86 | | -3,12 |
|  | **P8** | | 23,90 | 2,81 | 0,94 | | 23,23 | | 21,76 | | 10,63 | 23,81 | | 22,74 | | -4,94 | | 1,47 | 25,37 | | -4,09 | | -3,29 |
|  | **P9** | | 23,42 | 2,20 | 0,95 | | 22,26 | | 20,11 | | 12,10 | 60,65 | | 79,61 | | -1,77 | | 0,35 | 26,08 | | -0,46 | | -3,52 |
|  | **P20** | | 15,43 | 4,86 | 0,89 | | 15,10 | | 16,45 | | 5,93 | na | | na | | na | | na | 15,97 | | -11,26 | | -2,58 |
|  | **P21** | | 25,85 | 2,37 | 0,96 | | 24,20 | | 26,10 | | 14,67 | na | | na | | na | | na | 30,60 | | -0,85 | | -3,85 |
|  | **P22** | | 26,10 | 3,16 | 0,95 | | 24,26 | | 27,91 | | 11,56 | 23,68 | | 20,31 | | -8,24 | | 2,30 | 33,79 | | -2,80 | | -3,96 |
|  | **P29** | | 22,75 | 2,70 | 0,94 | | 22,32 | | 20,18 | | 10,65 | 22,17 | | 18,14 | | -6,49 | | 1,93 | 23,63 | | **-4,72** | | -3,16 |
|  | **P30** | | 19,84 | 1,85 | 0,95 | | 19,50 | | 17,16 | | 13,59 | na | | na | | na | | na | 20,46 | | **-0,28** | | -3,23 |
| Upper montane | **P10** | | na | na | na | | na | | na | | na | na | | na | | na | | na | na | | na | | na |
|  | **P11** | | na | na | na | | na | | na | | na | na | | na | | na | | na | na | | na | | na |
|  | **P12** | | 10,53 | 9,28 | 0,78 | | 10,49 | | 10,46 | | 3,25 | na | | na | | na | | na | 10,62 | | **-28,69** | | -1,66 |
|  | **P13** | | 11,95 | 2,46 | 0,89 | | 11,83 | | 11,20 | | 7,18 | na | | na | | na | | na | 12,13 | | -4,05 | | -2,46 |
| **Forest type** | | **Plot** | **Michaelis-Menten (m5)** | | | | | | | **Power (m6)** | | | | | | | **Second order polynomial (m7)** | | | | | | |
|  |  |  | **AIC** | | | **R²** | | **RSME** | | **AIC** | | | **R²** | | **RSME** | | **AIC** | | | **R²** | | **RSME** | |
| Sub montane | | **P23** | 689,6 | | | 0,805 | | 3,42 | | 695,1 | | | 0,79 | | 3,5 | | 688,7 | | | 0,81 | | 3,42 | |
|  |  | **P24** | 887,2 | | | 0,76 | | 4,56 | | 914,8 | | | 0,71 | | 5 | | 874,02 | | | 0,78 | | 4,58 | |
|  |  | **P25** | 1075,99 | | | 0,76 | | 4,26 | | 1101,6 | | | 0,72 | | 4,6 | | 1069,9 | | | 0,77 | | 4,2 | |
|  |  | **P26** | 691,01 | | | 0,775 | | 3,51 | | 711,1 | | | 0,737 | | 3,8 | | 689,04 | | | 0,78 | | 3,46 | |
|  |  | **P27** | 664,4 | | | 0,821 | | 2,36 | | 664,72 | | | 0,819 | | 2,38 | | 662,94 | | | 0,823 | | 2,35 | |
|  |  | **P28** | 669,93 | | | 0,697 | | 3,37 | | 682,8 | | | 0,664 | | 3,55 | | 672,27 | | | 0,696 | | 3,38 | |
| Lower montane | | **P14** | 661,47 | | | 0,764 | | 3,33 | | 682,17 | | | 0,7222 | | 3,62 | | 650,09 | | | 0,788 | | 3,16 | |
|  |  | **P15** | 703,86 | | | 0,847 | | 3,34 | | 724,79 | | | 0,821 | | 3,61 | | 693,53 | | | 0,861 | | 3,18 | |
|  |  | **P16** | 796,79 | | | 0,852 | | 2,96 | | 816,02 | | | 0,833 | | 3,14 | | 773,86 | | | 0,874 | | 2,73 | |
|  |  | **P17** | 809,28 | | | 0,872 | | 2,76 | | 837,56 | | | 0,848 | | 3,01 | | 804,3 | | | 0,877 | | 2,7 | |
|  |  | **P18** | 1256,2 | | | 0,759 | | 3,27 | | 1285,4 | | | 0,728 | | 3,48 | | 1241,8 | | | 0,775 | | 3,16 | |
|  |  | **P19** | 845,9 | | | 0,802 | | 3,18 | | 861,34 | | | 0,743 | | 3,62 | | 832,18 | | | 0,789 | | 3,28 | |
| Middle montane | | **P1** | **694,11** | | | **0,618** | | **2,67** | | 294,29 | | | 0,617 | | 2,67 | | 295,7 | | | 0,621 | | 2,73 | |
|  |  | **P2** | 367,92 | | | 0,75 | | 1,89 | | 387,17 | | | 0,689 | | 2,11 | | 366,64 | | | 0,759 | | 1,86 | |
|  |  | **P3** | 341,76 | | | 0,668 | | 3,33 | | 350,63 | | | 0,617 | | 3,57 | | 345,48 | | | 0,658 | | 3,38 | |
|  |  | **P4** | 718,21 | | | 0,737 | | 2,44 | | 731,97 | | | 0,713 | | 2,56 | | 706,5 | | | 0,759 | | 2,36 | |
|  |  | **P5** | 348,22 | | | 0,788 | | 2,23 | | 358,64 | | | 0,758 | | 2,39 | | 348,72 | | | 0,792 | | 2,21 | |
|  |  | **P6** | 898,31 | | | 0,66 | | 2,33 | | 919,51 | | | 0,622 | | 2,46 | | 887,74 | | | 0,68 | | 2,26 | |
|  |  | **P7** | 585,53 | | | 0,806 | | 2,07 | | 629,12 | | | 0,737 | | 2,41 | | 580,19 | | | 0,816 | | 2,01 | |
|  |  | **P8** | 910,24 | | | 0,762 | | 3,35 | | 950,47 | | | 0,699 | | 3,77 | | 898,95 | | | 0,788 | | 3,23 | |
|  |  | **P9** | **822,46** | | | **0,756** | | **2,75** | | 831,3 | | | 0,743 | | 2,82 | | 823,22 | | | 0,758 | | 2,76 | |
|  |  | **P20** | 558,29 | | | 0,694 | | 2,67 | | 580,99 | | | 0,687 | | 2,95 | | 523,47 | | | 0,78 | | 2,28 | |
|  |  | **P21** | **908,52** | | | **0,72** | | **3,34** | | 913,26 | | | 0,712 | | 3,38 | | 907,03 | | | 0,715 | | 3,33 | |
|  |  | **P22** | 452,84 | | | 0,788 | | 3,16 | | 455,98 | | | 0,78 | | 3,21 | | 451,9 | | | 0,795 | | 3,1 | |
|  |  | **P29** | 459,46 | | | 0,646 | | 3,49 | | 470,27 | | | 0,598 | | 3,71 | | 458,54 | | | 0,658 | | 3,43 | |
|  |  | **P30** | **261,25** | | | **0,78** | | **2,27** | | 266,34 | | | 0,745 | | 22,37 | | 260,01 | | | 0,781 | | 2,3 | |
| Upper montane | | **P10** | 361,12 | | | 0,524 | | 2,67 | | **357,27** | | | **0,548** | | **2,6** | | 360,97 | | | 0,538 | | 2,63 | |
|  |  | **P11** | 218,26 | | | 0,153 | | 2,11 | | **218,14** | | | **0,155** | | **2,11** | | 220,16 | | | 0,154 | | 2,11 | |
|  |  | **P12** | 229,17 | | | 0,569 | | 1,84 | | 234,36 | | | 0,529 | | 1,93 | | 226,65 | | | 0,602 | | 1,77 | |
|  |  | **P13** | 808,03 | | | 0,498 | | 2,16 | | 823,58 | | | 2,26 | | 0,454 | | 802,5 | | | 0,519 | | 2,12 | |
|  | |  | **a** | | | | | **b** | | **a** | | | | | **b** | | **a** | | | **b** | | **c** | |
| Sub montane | | **P23** | 79,50 | | | | | 122,10 | | 1,08 | | | | | 0,78 | | -3,49 | | | -1,14 | | 2,04 | |
|  |  | **P24** | 57,96 | | | | | 70,15 | | 1,91 | | | | | 0,64 | | -37,05 | | | 20,12 | | **-1,10** | |
|  |  | **P25** | 70,68 | | | | | 96,48 | | 1,35 | | | | | 0,73 | | -12,74 | | | 4,74 | | 1,22 | |
|  |  | **P26** | 48,73 | | | | | 63,51 | | 1,67 | | | | | 0,64 | | 1,67 | | | 0,64 | | 0,0002 | |
|  |  | **P27** | 52,43 | | | | | 70,11 | | 1,55 | | | | | 0,67 | | **5,99** | | | **-4,98** | | 2,3 | |
|  |  | **P28** | 51,59 | | | | | 69,60 | | 1,58 | | | | | 0,66 | | -9,04 | | | 3,96 | | 0,98 | |
| Lower montane | | **P14** | 51,30 | | | | | 65,55 | | 1,52 | | | | | 0,60 | | -30,26 | | | 17,46 | | **-1,04** | |
|  |  | **P15** | 85,50 | | | | | 109,89 | | 1,28 | | | | | 0,77 | | **-11,30** | | | **3,28** | | **1,67** | |
|  |  | **P16** | 106,40 | | | | | 151,44 | | 0,98 | | | | | 0,84 | | **-7,80** | | | **0,42** | | 2,16 | |
|  |  | **P17** | 72,59 | | | | | 111,84 | | 1,15 | | | | | 0,75 | | -2,27 | | | -1,67 | | 2,07 | |
|  |  | **P18** | 58,37 | | | | | 79,52 | | 1,29 | | | | | 0,73 | | -18,90 | | | 9,84 | | **0,19** | |
|  |  | **P19** | 70,02 | | | | | 98,07 | | 1,13 | | | | | 0,77 | | -20,18 | | | 9,80 | | 0,38 | |
| Middle montane | | **P1** | 33,98 | | | | | 34,73 | | 2,05 | | | | | 0,59 | | **-3,26** | | | **2,82** | | **0,81** | |
|  |  | **P2** | 23,73 | | | | | 20,55 | | 3,24 | | | | | 0,42 | | -21,31 | | | 16,03 | | -1,62 | |
|  |  | **P3** | 27,19 | | | | | 27,22 | | 3,76 | | | | | 0,38 | | -14,42 | | | 10,75 | | -0,66 | |
|  |  | **P4** | 38,67 | | | | | 48,26 | | 1,51 | | | | | 0,66 | | -25,28 | | | 16,28 | | -1,27 | |
|  |  | **P5** | 30,55 | | | | | 32,99 | | 2,31 | | | | | 0,52 | | -20,13 | | | 13,60 | | **-0,97** | |
|  |  | **P6** | 33,00 | | | | | 33,01 | | 2,06 | | | | | 0,59 | | -28,06 | | | 19,74 | | -2,00 | |
|  |  | **P7** | 28,58 | | | | | 28,18 | | 2,88 | | | | | 0,46 | | -20,45 | | | 14,53 | | -1,18 | |
|  |  | **P8** | 41,16 | | | | | 53,99 | | 1,92 | | | | | 0,58 | | -24,89 | | | 14,83 | | -0,85 | |
|  |  | **P9** | 40,11 | | | | | 50,97 | | 1,63 | | | | | 0,64 | | **-6,79** | | | **3,99** | | **0,72** | |
|  |  | **P20** | 29,40 | | | | | 45,02 | | 1,40 | | | | | 0,61 | | -46,12 | | | 29,20 | | -3,46 | |
|  |  | **P21** | 52,62 | | | | | 85,10 | | 1,13 | | | | | 0,72 | | **-5,53** | | | **1,73** | | **1,19** | |
|  |  | **P22** | 94,12 | | | | | 197,01 | | 0,64 | | | | | 0,87 | | **5,99** | | | **-7,31** | | 2,75 | |
|  |  | **P29** | 34,79 | | | | | 39,93 | | 2,58 | | | | | 0,50 | | -31,06 | | | 18,72 | | -1,48 | |
|  |  | **P30** | 27,87 | | | | | 30,46 | | 2,46 | | | | | 0,49 | | -16,19 | | | 11,28 | | **-0,70** | |
| Upper montane | | **P10** | 34,47 | | | | | 52,12 | | 1,36 | | | | | 0,65 | | **5,05** | | | **-3,43** | | **1,64** | |
|  |  | **P11** | 10,71 | | | | | 11,36 | | 2,07 | | | | | 0,39 | | **1,05** | | | **1,23** | | **0,23** | |
|  |  | **P12** | 14,43 | | | | | 13,99 | | 2,83 | | | | | 0,35 | | -20,35 | | | 15,55 | | -1,90 | |
|  |  | **P13** | 16,48 | | | | | 17,32 | | 2,70 | | | | | 0,39 | | -20,72 | | | 15,32 | | -1,78 | |
