# Supplementary material for: Height-diameter allometry and above ground biomass in tropical montane forests: Insights from the Albertine Rift in Africa
Source: PLoS One. 2017 Jun 15;12(6):e0179653. doi: 10.1371/journal.pone.0179653 (PMC5472301; doi:10.1371/journal.pone.0179653)
Supplement: S2 Table — (DOCX) [file pone.0179653.s002.docx]

**S2 Table.** Correlation between soil attributes.

| Attributes | pH | H+ | Al^3+^ | K | | P | C | N | C/N | Clay | Sand | Silt |
| --- | --- | --- | --- | --- | --- | --- | --- | --- | --- | --- | --- | --- |
| pH | **1.00***** |  |  |  | |  |  |  |  |  |  |  |
| H+ | -0.41 | **1.00***** |  |  | |  |  |  |  |  |  |  |
| Al^3+^ | **-0.66***** | **0.50**** | **1.00***** |  | |  |  |  |  |  |  |  |
| K | 0.17 | 0.01 | -0.16 | **1.00***** | |  |  |  |  |  |  |  |
| P | 0.30 | 0.32 | -0.06 | 0.31 | | **1.00***** |  |  |  |  |  |  |
| C | 0.07 | 0.10 | **0.59**** | -0.08 | | 0.01 | **1,00***** |  |  |  |  |  |
| N | 0.08 | 0.13 | **0.52**** | -0.03 | | 0.13 | **0,81***** | **1,00***** |  |  |  |  |
| C/N | -0.09 | 0.08 | 0.13 | 0.03 | | -0.19 | 0,30 | -0,24 | **1,00***** |  |  |  |
| Clay | -0.08 | -0.17 | -**0.49**** | -0.30 | | -0.36 | **-0,70***** | **-0,77***** | 0,01 | **1,00***** |  |  |
| Sand | 0.21 | -1.12 | 0.14 | 0.21 | | 0.22 | 0,37 | 0,27 | 0,05 | **-0,54**** | **1,00***** |  |
| Silt | -0.13 | 0.31 | 0.37 | 0.10 | | 0.15 | 0,37 | **0,54**** | -0,07 | **-0,51**** | -0,45 | **1,00***** |
| Bulk density | 0.25 | -0.46 | **-0.72***** | 0.10 | | -0.07 | **-0,71***** | **-0,59***** | -0,21 | **0,53**** | -0,21 | -0,35 |
| CEC | -0.15 | 0.27 | **0.59***** | -0.09 | | 0.07 | **0,74***** | **0,65***** | 0,19 | **-0,57***** | 0,37 | 0,23 |
| Slope | -0.13 | -0.16 | -0.20 | -0.02 | | -0.22 | -0,23 | -0,26 | 0,14 | 0,11 | <0,01 | -0,11 |
| Species richness | 0.02 | -0.09 | **-0.48**** | 0.13 | | -0.11 | **-0,69***** | **-0,48**** | -0,46 | **0,54***** | -0,20 | -0,38 |
| Stem density | 0.10 | - 0.14 | 0.16 | -0.11 | | -0.39 | 0,40 | 0,47 | <0,01 | -0,22 | -0,22 | 0,45 |
| Altitude | -0.20 | 0.08 | **0.65***** | -0.10 | | -0.07 | **0,70***** | **0,52**** | 0,37 | **-0,56***** | 0,15 | 0,44 |
| Attributes | Bulk density | | | | CEC | | Slope | | Species richness | | Stem density | |
| Bulk density | **1.00***** | | | |  | |  | |  | |  | |
| CEC | **-0.71***** | | | | **1.00***** | |  | |  | |  | |
| Slope | 0.31 | | | | -0.22 | | **1.00***** | |  | |  | |
| Species richness | 0.48 | | | | **-0.60***** | | 0.18 | | **1.00***** | |  | |
| Stem density | -0.33 | | | | 0.29 | | -0.01 | | -0.25 | | **1.00***** | |
| Altitude | **-0.57***** | | | | **0.62***** | | -0.09 | | **-0.82***** | | **0.47**** | |

Significant correlations at p<0.01 as ** and at p < 0.001 as ***. The “r” of significant Pearson correlation is shown in bold.
