# Supplementary material for: Height-diameter allometry and above ground biomass in tropical montane forests: Insights from the Albertine Rift in Africa
Source: PLoS One. 2017 Jun 15;12(6):e0179653. doi: 10.1371/journal.pone.0179653 (PMC5472301; doi:10.1371/journal.pone.0179653)
Supplement: S3 Table — (DOCX) [file pone.0179653.s003.docx]

**S3 Table.** Soil main characteristics per forest type.

| **Forest type** | **pH (H_2_O)** |  | **C%** | | **N%** | | **C:N ratio** | | **CEC cmol+ kg-1** | | **BD g cm-3** | | **% Clay** | | **% Sand** | | **% Silt** | | **H+**  **(cmolc kg-1)** |  | **Total P mgP kg-1** | | **Al^3+^ cmolc kg-1** | | **K^+^ me g-100 soil** | |
| --- | --- | --- | --- | --- | --- | --- | --- | --- | --- | --- | --- | --- | --- | --- | --- | --- | --- | --- | --- | --- | --- | --- | --- | --- | --- | --- |
| Sub montane | 4,6 ± 0.33 | a | 2,17 ± 0,62 | a | 0,3 ± 0,11 | a | 7,4 ± 1,8 | a | 13 ± 2,02 | a | 1,15 ± 0,17 | a | 40,9 ±13,8 | a | 37,14 ± 11,02 | a | 21,99 ± 11,7 | a | 1.25 ± 0.17 | a | 28,5 ± 10,1 | a | 3.05 ± 1.4 | a | 0,2 ± 0,07 | a |
| Lower montane | 4,08 ± 0.15 | b | 1,92 ± 0,4 | a | 0,24 ± 0,03 | a | 8,2 ± 2,8 | a | 14,03 ± 4,8 | ab | 1,03 ± 0,13 | a | 47,2 ± 3,4 | a | 35,8 ± 5,5 | a | 17 ± 2,1 | a | 1.22 ± 0.14 | a | 13,03 ± 4,08 | b | 5.37 ± 1.17 | b | 0,14 ± 0,01 | ab |
| Middle montane | 4.21 ± 0.32 | ab | 3,27 ± 1,35 | ab | 0,32 ± 0,14 | ab | 10,7 ± 3,3 | a | 22 ± 9,4 | ab | 0,98 ± 0,23 | a | 36,6 ± 10,7 | ab | 37,9 ± 13,4 | a | 25,6 ± 10,1 | a | 1.24 ± 0.22 | a | 21,3 ± 10,01 | b | 6.02 ± 2.62 | b | 0,19 ± 0,03 | a |
| Upper montane | 4.4 ± 0.41 | ab | 5,3 ± 1,03 | b | 0,55 ± 0,1 | b | 9,7 ± 2,6 | a | 23,8 ± 4,8 | b | 0,84 ± 0,06 | a | 25,4 ± 8,2 | b | 40,6 ± 13,2 | a | 34 ± 15,2 | a | 1.24 ± 0.22 | a | 18,3 ± 8,45 | b | 7.73 ± 1.35 | c | 0,13 ± 0,03 | b |

Different letters within columns mark significant differences at p<0.05.
