# Supplementary figures and images for: Height-diameter allometry and above ground biomass in tropical montane forests: Insights from the Albertine Rift in Africa
Source: PLoS One. 2017 Jun 15;12(6):e0179653. doi: 10.1371/journal.pone.0179653 (PMC5472301; doi:10.1371/journal.pone.0179653)

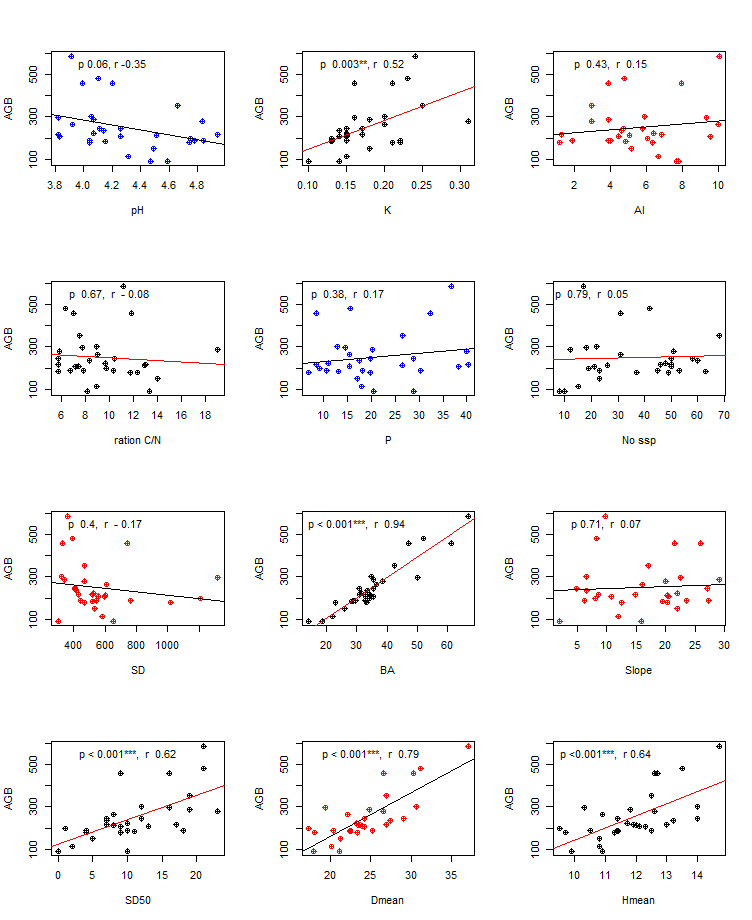

Supplement: S1 Fig — (TIF) [file pone.0179653.s004.tif]
